# An overview of Ehlers Danlos syndrome and the link between postural orthostatic tachycardia syndrome and gastrointestinal symptoms with a focus on gastroparesis

**DOI:** 10.3389/fneur.2024.1379646

**Published:** 2024-08-29

**Authors:** William Wu, Vincent Ho

**Affiliations:** ^1^Department of Gastroenterology, Campbelltown Hospital, Campbelltown, NSW, Australia; ^2^School of Medicine, Western Sydney University, Campbelltown, NSW, Australia

**Keywords:** ehlers danlos syndrome, postural orthostatic tachycardia syndrome, gastroparesis, autonomic dysfunction, astrointestinal dysfunction

## Abstract

There has been an increasingly reported association between Ehlers-Danlos syndrome (EDS), postural orthostatic tachycardia syndrome (POTS) and gastrointestinal disorders. EDS is a hereditary connective tissue disorder which may manifest as a spectrum of symptoms stemming from collagen defects. The prevalence of EDS is estimated to affect 1 in 5000 individuals which underscores its clinical significance. Notably the hypermobile form (hEDS) accounts for the majority of cases. POTS is characterized by orthostatic intolerance with an increase in heart rate on standing in the absence of hypotension. This condition predominantly affects women between 15 and 45 years of age. Gastrointestinal symptoms in the form of reflux, bloating and abdominal pain significant impact this population. Gastroparesis is a chronic disorder involving symptoms of delayed gastric emptying and may be closely associated with hEDS and POTS, and may be underreported. Autonomic dysfunction associated with hEDS has been proposed as the likely mechanism underlying POTS and gastrointestinal dysfunction though a clear pathophysiological process has not been established.

## 1 Introduction

Ehlers-Danlos syndrome (EDS) constitutes a collection of hereditary and heterogenous connective tissue disorders impacting the skin, ligaments, joints, blood vessels and internal organs (1). The prevalence of EDS is estimated at approximately 1 in 5000 individuals (2). The primary issue arises from defects in collagen which is the principal structural component of connective tissues. Clinical manifestations of EDS encompass a spectrum of features, ranging from skin fragility, hyperextensible skin, joint hypermobility, excessive bruising, and atypical scarring to severe vascular complications (1). There at 13 subtypes of EDS of which the hypermobile form (hEDS) is most prevalent accounting for 80–90% of cases of EDS with several subtypes exhibiting significant symptom overlap (3). The dysregulation of connective tissue in patients with EDS may impact the cardiovascular, gastrointestinal, immune, and autonomic systems affecting structure and function (4–6). A significant proportion of patients with EDS experience troubling gastrointestinal symptoms with up to 98% fulfilling Rome IV criteria for a functional gastrointestinal disorder (7). Gastroparesis in EDS is less well defined within the spectrum of associated gastrointestinal symptoms.

Postural orthostatic tachycardia syndrome (POTS) is a heterogeneous condition which is characterized by an increase in heart rate of >/= 30 bpm that occurs within 10 min of standing. There is an absence of orthostatic hypotension that is defined as a decrease in systolic blood pressure > 20 mmHg or diastolic blood pressure > 10 mmHg (8). It is more common in women compared with men with a ratio of at least 4.5:1 and a range between15-45 years of age (8, 9). POTS is characterized by symptoms of light-headedness, mental fogging, blurred vision, dyspnoea and palpitations (8). Incumbering gastrointestinal symptoms such as reflux, nausea, bloating, abdominal pain and altered bowel habits are the most prominent non-cardiovascular complaints among this population (10).

Gastroparesis is a chronic disorder that is characterized by symptoms of delayed gastric emptying in the absence of mechanical obstruction (11). While a diagnosis is often accompanied by functional investigations such as gastric emptying studies, these tests are imperfect due to patient variables and a lack of standardized cut-offs. Additionally, it can be difficult to differentiate gastroparesis from functional dyspepsia based on clinical features (12). There may be a higher than previously reported incidence of gastroparesis in patients with EDS and POTS.

There is an increased prevalence of autonomic dysfunction in patients with EDS with reportedly up to 40% having some disorder of orthostatic intolerance such as POTS (8). Conversely up to 18% of patients with POTS have been found to have EDS (13). Patients with both conditions appear to also have increased symptom burden (14, 15). The underlying mechanism remains unclear though there are hypotheses underscoring the pathophysiology based off structural and functional abnormalities. This review article aims to highlight the association between EDS, POTS and functional gut disorders with a focus on gastroparesis. Increased awareness and recognition of associated symptoms may lead to more timely and accurate diagnoses which in turn reduces delays in management leading to overall improved patient care.

## 2 Overview of EDS

### 2.1 Genetics of EDS

The hereditable genetic mutations underlying most forms of EDS have been studied and identified. These alterations to various genes disrupt collagen production, enzymes responsible for modifying collagen or proteins influencing collagen fibrillogenesis and structure. This disruption leads to abnormalities such as fibril disorganization, altered bundle size or reduced collagen synthesis (3, 16). Mutations in the COL5A1 and COL5A2 genes, which encode the α1 and α2 chains of type V collagen, are found in about 50% of individuals clinically diagnosed with classic EDS. In roughly one-third of these cases, the condition is due to a mutation that produces a non-functional COL5A1 allele, resulting in haploinsufficiency of type V collagen (17). However, a genetic defect underlying the most common form being hEDS has not been definitively established and it has been speculated that genes other than those involved in encoding collagen and collagen modifying enzymes are involved (16). The ability to establish a confirmative molecular diagnosis for hEDS would be ideal given the significant heterogeneity with other clinical syndromes which lead to delayed or inaccurate diagnosis.

### 2.2 Sex discrepancy in the etiology of hEDS

Despite the presumed autosomal dominant inheritance pattern of hEDS, there is a well-documented female predominance of at least 2:1 and reports of up to 9:1 in some groups (18). There appears to be a divergence from adolescence as the ratio in early childhood is more similar (19), which does suggest a strong hormonal influence. In the general population, ligament laxity has been shown to be influenced by hormones such as estrogen, progesterone, relaxin, and testosterone, with the most extensive research conducted in the context of anterior cruciate ligament (ACL) injuries in women. The risk of ACL injury and knee ligament laxity tends to be higher during the preovulatory and ovulatory phases of the menstrual cycle, when estrogen levels surpass those of progesterone. Hormonal contraceptives have been suggested to play a potentially protective role in preventing ACL tears. The observed effects of hormones on ligament laxity, along with patient-reported symptom fluctuations that align with hormonal changes, underscore the need for further research to clarify the role of hormones in hEDS (20). There are likely other contributing factors which others have explored including; greater muscle mass and ligamentous stiffness in men and a greater propensity for women to seek medical attention earlier (21). Further studies looking into protective mechanisms in men could certainly be of value to treating patients with hEDS (20).

### 2.3 Diagnostic criteria for hEDS

The Beighton scoring system for assessing joint hypermobility has been adopted as a clinical tool for the diagnosis of hEDS. The criteria is scored out of 9 and tests for hypermobility of joints in the upper and lower limbs as well as the spine. Apposition of the thumb to the forearm, hyperextension of the 5th finger beyond 90o, hyperextension of the elbows and knees and forward flexion of the trunk (22). The Beighton scoring system was initially designed as a screening tool and its ongoing use as a diagnostic tool remains somewhat controversial. The main opponents argue that too few joints are tested, patients with borderline hypermobility of tested joints are underrepresented and it is unable to account for intrapatient variability (23). As with all scoring systems appropriate clinical judgment should be implemented by the clinician to ensure diagnostic accuracy.

### 2.4 Diagnostic challenges

The diagnosis for patients with EDS and subtypes is difficult due to the variability in presentations with less than half of patients being diagnosed before the age of 30 (24). A European survey of patient’s rare genetic diseases concluded that patients with EDS had the longest delays in diagnosis often transitioning through multiple specialists (25). Delays in diagnosis and misdiagnoses are common and consequently contributes to a significant burden and impact on quality of life (26).

### 2.5 Clinical course of hEDS

The manifestations of hEDS are dynamic and a progression of the disorder has previously been described. Hyperflexible joints and recurrent subluxations/dislocations with or without pain are generally seen in an adolescent population. Musculoskeletal pain may worsen in the early adult years with lessened hypermobility. This may develop further into adult hood as chronic fatigue and pain with further limited hypermobility features (18). This progressive nature of hEDS is somewhat accounted for in the diagnostic algorithm where a reduced Beighten score is required for a diagnosis beyond the age of 50 (23). An awareness of this shift in signs is an important nuance for clinicians to be aware of.

### 2.6 Manifestations of EDS

EDS is a heterogeneous disorder that may affect multiple organ systems, however for the purposes of this article, there will be a focus on the following:

#### 2.6.1 Dysautonomia

Autonomic dysfunction in patients with hEDS may present as tachycardia, postural hypotension, gut dysmotility, disrupted bladder function and altered regulation of sweating (20). A subset of patients with particularly disruptive symptoms may have POTS which has previously been established as being closely associated with hEDS as well as generalized joint hypermobility not meeting criteria for hEDS (27). Peripheral venous dilation with blood pooling and increased circulating catecholamines provide potential mechanisms underlying cardiovascular dysautonomia (5), the possible pathophysiologic processes will be discussed further on.

#### 2.6.2 Gastrointestinal symptoms

Gastrointestinal symptoms of abdominal pain, bloating, nausea, reflux, vomiting and altering bowel habits are common issues in patients with EDS (6). The underlying pathophysiology likely relates to structural and functional issues for which several theories have been hypothesized (28–30). There is a growing emphasis on dysautonomia as a contributor to the development and progression of gastrointestinal issues (31). The diagnostic criteria for hEDS do not include gastrointestinal symptoms despite the high prevalence in this population. Clinicians should be well aware of gastrointestinal symptoms despite this. There is a lack of guidelines specific to gastrointestinal dysfunction in EDS at present and management is generally targeted to a specific symptom (20).

### 2.7 Treatment of hEDS

There is no targeted therapy for patients with hEDS, rather treatment is aimed at symptoms and complications of the disease process. The management of pain is the same as what would be utilized in a general population with a combination of pharmacological and non-pharmacological therapies (32). Fatigue is difficult to tackle in this population and the predominant treatment focuses on lifestyle modifications. The management of autonomic dysfunction, predominantly cardiovascular and gastrointestinal disorders will be discussed further on. Due to the multi-system involvement of this condition, the management of patients with hEDS should involve a multidisciplinary team.

## 3 Overview of POTS

### 3.1 Clinical features of POTS

Patients with POTS typically present between 15 and 50 years of age, and as previously mentioned, there is a strong female predominance (33). The postural symptoms of light-headedness, mental fogging, blurred vision, dyspnoea and palpitations may be accompanied by gastrointestinal disturbances, fatigue, sleep disturbance and migraine (10). Progression in severity of symptoms have been reported by patients as they age (34). In addition, factors such as hydration, temperature, humidity, stage of menstrual cycle can affect symptoms (8).

### 3.2 Diagnostic criteria for POTS

There is no universally accepted set of criteria for the diagnosis of POTS, although Olshanksy et al. (35) have suggested the following:

Reproducible orthostatic tachycardia with a rise in heart rate >/= 30 bpm for those >19 years of age with symptoms of orthostatic intolerance.

1. A clear definition of orthostatic change in position and time in each position.
2. Orthostatic tachycardia within 3–10 min of standing and/or on a tilt table test.
3. No evidence of orthostatic hypotension at any time with standing.
4. A chronic condition present for at least 6 months.
5. No other explainable cause for orthostatic tachycardia or tachycardia.
6. Symptoms of orthostatic intolerance that include postural chest pain, exertional dyspnoea, dependent acrocyanosis, dizziness, light-headedness with associated heart rate abnormalities.
7. Orthostatic symptoms disappear when supine.
8. Extra orthostatic symptoms – chronic fatigue, “brain fog.”
9. Other autonomic symptoms – bloating, constipation, sweating abnormalities.
10. Syncope is not a criterion.
11. Symptoms alone do not make the diagnosis.
12. “Secondary” orthostatic tachycardia is not POTS.

### 3.3 Diagnostic challenges for POTS

The presence of numerous, severe symptoms causing substantial disability in the absence of an identifiable cause often prompts consideration of a psychogenic origin. This controversy surrounding the nature of POTS has resulted in diagnostic confusion, leading to the inappropriate use of testing and treatment strategies (36). Patients with POTS may experience significant disability and impact on quality of life with data suggesting 25% of patients file for disability and greater than 50% have educational interruptions (37). Delays in diagnosis may further impact upon patient welfare.

On the other hand, an increasing incidence of patients are labeling themselves with a diagnosis of POTS based on vague symptoms, probably as a result of the increasing amount of accessible information and misinformation (38).

The Hearth Rhythm society has published recommendations regarding the suggested workup of patients suspected to have POTS (39). Performing a thorough history and examination with orthostatic vital monitoring and 12-lead ECG has the highest recommended tier followed by a complete blood count with assessment of thyroid function. Investigations such as 24-h Holter monitoring, transthoracic echocardiogram, tilt-table testing and exercise tolerance testing can be considered in select patients.

### 3.4 Proposed mechanisms underlying POTS

The underlying pathology for POTS remains elusive. Several mechanisms have been proposed as the potential pathophysiology behind POTS and are often labeled as the sub-types of neuropathic, hyperadrenergic and hypovolemic POTS (40).

Neuropathic POTS suggests that there is an underlying partial sympathetic neuropathy with a length dependent distribution where there is a blunted vasoconstriction in response to stimuli causing venous congestion, particularly in the lower limbs (41). This theory is supported by measurements of reduced sympathetic firing in the legs compared with the arms (42). An increase in heart rate results from compensatory feedback mechanisms (40).

Hyperadrenergic POTS explores an excess of plasma norepinephrine release and rise in blood pressure with standing. Support for this theory comes from a study which demonstrated elevated plasma norepinephrine levels on standing in 29% of a cohort of 152 patients (34).

Patients with POTS have been observed in a small study of 29 patients to have reduced plasma and red cell volume compared with healthy controls. Interestingly these patients also had lower levels of aldosterone and inappropriately lower levels of plasma renin in the context of their degree of hypovolemia, suggesting that the exocrine function of the kidney may be involved (43).

### 3.5 Outline of management of POTS

There is no specific targeted treatment for an entity, nor is there a cure for POTS. Treatment is aimed at relieving symptoms associated with POTS and reducing burden of disease.

Non-pharmacological approaches are generally suggested as a first line approach, these measures include removing medications contributing to symptoms if possible, ensuring adequate hydration and increasing salt intake (40). There are several pharmacological options for specific symptoms, although none are FDA approved (44).

## 4 Overview of gastroparesis

### 4.1 Normal gastric function

Understanding the complexities of gastric functions and how dysfunction is manifested through clinical symptoms is important for precise diagnosis of gastroparesis and effective therapeutic interventions. Normal stomach function involves dynamic interplay of physiological process crucial for digestive functions. This intricate regulatory network involves fundal relaxation, antral grinding, trituration and propulsion. This is modulated by intrinsic and extrinsic factors including gut hormones, the gastric pacemaker, and higher cerebral centers. Meal characteristics, such as composition and physical nature, exert notable influences on this process. Furthermore, gastric pump failure can emanate from gastric dysrhythmias, impaired compliance, and gastric outlet obstruction (45).

The gastric emptying process involves complex interactions within distinct stomach chambers and the duodenum. The fundus serves to mediated intragastric pressure and triggers tonic propulsion of chyme into the distal stomach, and this modulated by enteric and hormonal influences (46). The proximal stomach also functions to accommodate meals and provide temporary storage. This accommodation can support over 1 L of contents without an increase to intragastric pressure. This is facilitated through receptive relaxation and gastric accommodation reflexes which lead to a drop in proximal gastric tone in response to swallowing contents (45, 47). Gastric accommodation is also supported by reflexive relaxation is also a component of gastric accommodation whereby neurohormones are released in response to increase in gastric contents (48). This is a vagally mediated response which provides some explanation for the reduced gastric distensibility and increased intragastric pressures following a bolus in patients with a vagotomy (49). Finally, there is the enterogastric reflex by which exposure of proteins or lipids in the small bowel affects proximal gastric motor activity, and this feature also appears to be vagally driven (50).

Activity in the distal stomach is characterized by phasic motor activity. This can be observed during endoscopy as a peristatic wave which propagates from the distal body and terminates at the pylorus. This rhythmic movement is due to phasic depolarisation of intestinal cells of Cajal termed the gastric slow wave (51). Propagation of the slow wave into the fundus is inhibited by the relatively more negative resting membrane potential in the proximal stomach. The slow wave amplitude may be influenced by neurohormonal triggers which in turn alter peristaltic activity (45, 52).

An understanding of gastric dynamics establishes a foundation for grappling the complexities inherent in gastric motility disorders such as gastroparesis. Poor glycaemic control in patients with diabetes, post-surgical complications and are well established aetiologies of gastroparesis. However there remains a larger proportion of patients with gastroparesis which is deemed idiopathic (53). Gastric dysmotility is associated with conditions such as EDS and POTS which occur from disruptions to normal gastric function.

### 4.2 Pathophysiology

Gastric biopsies from patients with gastroparesis reveal a paucity of Cajal cells. Furthermore, patients with absence of Cajal cells appear to demonstrate increase severity of gastrointestinal symptoms. This subgroup of patients tend to have significant abnormalities with electrogastrography and response poorly to gastric electrical stimulation (54). Gastroparesis may present acutely in some patients following a viral-like gastroenteritis which raises some speculation regarding a viral etiology of idiopathic gastroparesis (55). In a similar nature, a viral trigger has also been speculated in the development of POTS.

### 4.3 Diagnostic modalities

Gastric emptying scintigraphy remains the current gold standard for evaluation of gastric dysmotility. Delayed gastric emptying has been defined as >10% retention in the stomach at 4 h while rapid gastric emptying is defined as >30% at 1 h (56). Evaluating solid emptying through scintigraphy over a 4-h period proves to be a more sensitive test, featuring defined normal ranges. The proportion retained at 2 and 4 h demonstrates a sensitivity of 90% and a specificity of 70% in identifying delayed emptying (57). It should be noted that diagnostic criteria for gastric emptying studies are not standardized between studies.

### 4.4 Diagnostic challenges

The diagnosis of functional dyspepsia and gastroparesis is difficult to differentiate based on clinical features and pathologies, even with the use of gastric emptying studies due to high inter- and intraindividual coefficient variation for gastric emptying (12). This underscores the critical need for accurate diagnosis using optimal measurement of gastric emptying through scintigraphy and the application of robust normative data. Strict cutoff criteria, such as gastric retention exceeding 75% at 2 h and over 25% at 4 h are particularly important (58). This is compounded by the lack of standardized figures between studies as previously mentioned.

Diagnostic testing is predominantly directed by the pattern and severity of symptoms. While scintigraphy and stable isotope breath tests can confirm delayed gastric emptying, the presence of retained food observed during endoscopy has limited predictive value, particularly unless the patient has a known underlying condition predisposing them to gastric retention. Barium studies and scintigraphy utilizing labeled liquid or semi-solid meals are typically unremarkable and offer limited diagnostic utility, even in cases with moderately severe symptoms (11).

As established, gastrointestinal symptoms are very common in patients with EDS with up to 50% having been diagnosed with functional dyspepsia or IBS. There remains difficulty in establishing a diagnosis of gastroparesis, as limited studies have been done to investigate whether a subset of these patients may actually have gastroparesis or other dysmotility.

### 4.5 Outline of management of gastroparesis

#### 4.5.1 Dietary modifications

Dietary modifications are a reasonable initial approach in managing gastroparesis. The risk of nutritional deficiencies is higher in patients with gastroparesis due to the tendency to restrict food options to a limited number of food or food groups (45). Excess fat and fiber intake lead to increased gastric emptying times and reduction in these food groups is recommended. Alterations to meal content and increased frequency with reduced portions may provide a risk-free intervention in improving nutrition in these patients (10, 59). The addition of non-solid supplements may help support nutritional deficits.

Glycaemic control should be optimized to mitigate further progression of delayed gastric emptying (60). Patients should be counseled to abstain from alcohol and cigarettes.

The symptoms of gastroparesis may result in reduced oral and fluid intake or increased gastrointestinal losses. These symptoms may well result dehydration, malnourishment and electrolyte disturbances which directly affect the core treatment of POTS.

#### 4.5.2 Prokinetics

Prokinetic medications increase antral contractility, reduce gastric dysrhythmias and improves coordination between antrum and duodenum. The effect is able to be measured with improvement in gastric emptying studies, although this does not correlate consistently with improvement in symptoms (45, 61). Interestingly, there appears to be a placebo effect on symptomatic improvement in patients with non-ulcer related dyspepsia (62).

#### 4.5.3 Antiemetics

Nausea and vomiting stand out as the most incapacitating symptoms of gastric pump failure. Antiemetics may be employed independently or along with prokinetics. Although specific antiemetic regimens for gastroparesis lack robust support from trials, empirical choices base on clinical experience suggest that a variety of antiemetics could be beneficial in managing nausea and vomiting (45).

#### 4.5.4 Antidepressants

While tricyclic antidepressants generally hinder gastrointestinal motility due to their cholinergic properties, there is evidence to support low-doses as a neuromodulator to provide relief with nausea, vomiting and pain (63). Patients should be made aware of infrequent side effects including sedation and dry mouth.

Amitriptyline has been demonstrated to improve antroduodenal motility in patients with POTS and symptomatic gastroparesis though tangible symptomatic improvement is unclear (64).

#### 4.5.5 Pyloric injection of botulinum toxin

Botulinum toxin A acts by inhibiting acetylcholine release leading to temporary muscle paralysis as a means to limit pylorospasm. Its intramuscular injection to the pylorus during endoscopy ideally has effects lasting for months with gradual restoration of function requiring repeated injections. The efficacy of botulinum is debatable with non-consistent data across studies, though a there are clinical trial data suggesting no benefit over placebo (65, 66).

#### 4.5.6 Enteral nutrition

Enteral feeding should be implemented if patients are unable to sustain their weight despite conventional interventions. A nasojejunal tube may be trialed to bypass the stomach and direct nutrition directly to the small bowel. Provided there is a satisfactory response, enteral feeding can also be implemented through a percutaneous gastrotomy with jejunostomy or a surgical jejunostomy (67).

#### 4.5.7 Surgery

Gastrectomy has been utilized in cases of severe, refractory gastroparesis (68). There is obviously a substantial risk with the intervention, limited data suggest that a highly selected cohort may benefit from the procedure (69, 70).

A systematic review of gastrointestinal surgeries in patients with EDS suggests a higher risk of complications such as incisional hernias and poor wound healing. This is likely due to collagen dysregulation affecting ligaments, blood vessels and viscera (71).

## 5 Overlap between EDS, POTS and gastrointestinal symptoms

### 5.1 Autonomic dysfunction underscoring

#### 5.1.1 Pots

The presence of systemic autonomic dysfunction in patients with hEDS are well documented, however an exact pathophysiological process has not been established. A recurrent association of POTS which was first described in 1999 (72) in patients with hEDS has lead to a prevailing theory of dysautonomia as a primary etiology (40). In particular, cardiovascular dysautonomia and sudomotor dysfunction which account for symptoms of orthostatic intolerance, light headedness, palpitations, chest pains, reduced sweat production and fatigue.

Furthermore, laxity in connective tissue in hEDS patients may result in increased venous compliance and arterial elasticity leading to a blood pooling and impaired vasoconstrictive response when standing (5, 27).

#### 5.1.2 Gastrointestinal symptoms

An increased prevalence of gastrointestinal symptoms in hEDS was first described in 2004 (73). Due to the close association with POTS and the overlapping gastrointestinal issues common between the two conditions, autonomic dysfunction has thus also been speculated to be a primary culprit (28).

A proposed mechanism for the prevalence of gastrointestinal symptoms is the lack of response to sympathetic stimulation which usually provides an inhibitory response to the enteric nervous system (10). The dysregulation to the sympathetic inhibitory response may lead to uncoordinated gastrointestinal activity (74).

Worsening gastrointestinal symptoms post-prandially has been reported in a POTS population (10). One suggested mechanism for this involves splanchnic vessel capacitance. The splanchnic vascular compartment receives up to 25% of the resting cardiac output in healthy individuals (75). The splanchnic circulation has a large capacitance and thus has a role during postural changes and after meals. Mesenteric blood volume may increase up to 300% following a standard meal which is mediated through release of vasoactive gut hormones (75, 76). Patients with POTS have been demonstrated to have a greater resting and greater increase in post-prandial mesenteric blood flow when compared with controls suggesting greater splanchnic capacitance (77). Impaired systemic sympathetic response to oppose this greater capacitance could explain the post-prandial tolerances of these patients.

Greater fluctuations in gastric electrical activity have been observed in patients with POTS in pre- and post-prandial states. Greater variability in electrical activity were seen in patients with gastrointestinal symptoms (78). Furthermore, significantly more gastric arrythmias have also been observed in POTS patients undergoing tilt table testing (79). The gastrointestinal symptoms in POTS may certainly be explained in some degree by gastric arrhythmias and is an aspect that has received limited exploration but holds potential significance.

In patients with severe autonomic failure, there is a lack of vasoconstrictive response with sympathetic stimulation to the superior mesenteric artery which may contribute to post-prandial hypotension (80). The exacerbation in symptoms and haemodynamic post-prandially in patients with POTS may further be explained by similar mechanisms given that the underlying pathology may be related to autonomic dysregulation.

### 5.2 Morphological gastrointestinal abnormalities in patients with hEDS

An increased propensity for morphological abnormalities such as abdominal hernias and rectal prolapse in hypermobility disorders have been investigated, although a tangible correlation is not apparent (81, 82). A rationale for these issues would be attributed to structural changes in collagen supporting the gastrointestinal system. Connective tissue is strongly represented in various components of the apparatus, such as peritoneal ligaments, the gut wall and splanchnic vessels. Symptoms may certainly arise from increased abdominal visceral mobility due to laxity of peritoneal ligaments (83).

Abnormalities to the connective tissue composition of gastric mucosa can impact the stomachs functionality by augmenting the compliance leading to excessive distension (84). Additionally, it can directly disrupt gut mechno-receptors within the muscularis externa. Increased permeability of gut mucosa may could also be driven by defects of the extracellular matrix (85). The cumulative effects of these phenomenon may extend to influencing pain thresholds and gut motility driving the gastrointestinal symptoms in patients with hEDS.

Patients with EDS have cutaneous and oral mucosal disruption in part from capillary fragility. Precise data is not available for whether this extends throughout the rest of the gastrointestinal tract. Although diminished resilience in capillaries and small vessels might play a role in peripheral blood steal thus exacerbating symptoms of nausea and bloating (84).

### 5.3 A focus on gastroparesis

While several primary studies have reported on the prevalence of gastrointestinal symptoms in patients with hEDS and POTS. These symptoms are also common in patients with gastroparesis, however there are limited studies which further investigate these symptoms with gastric motility studies. The largest study included a cohort of 687 patients over a 20-year period where gastric emptying studies were performed on 76 patients. Abnormal emptying was observed in 17 patients with 9 having delayed emptying (30). A smaller study involving 218 hEDS patients found 12 of 26 tested patients had delayed gastric emptying (24). While the apparent incidence of delayed emptying is highly variable between the two studies, the incidence is far higher than the estimated 21.5 per 100,000 in a general population (86). More data are needed to assess gastric dysmotility in patients with hEDS.

## 6 Conclusion

In conclusion, this review provides an overview of hEDS, POTS, and the gastrointestinal issues commonly associated with both conditions, particularly gastroparesis. While there is a notable overlap between these conditions, no definitive pathophysiological mechanism has been identified to link them, and the underlying mechanisms for each condition remain uncertain. The etiology of hEDS remains unclear, with no specific genetic mutation yet established. The heterogeneous presentation of hEDS, with overlapping signs and symptoms across different conditions, underscores the need for further research to establish a molecular diagnosis, which could help reduce delays in patient management.

Diagnosing POTS is also challenged by the absence of universally accepted diagnostic criteria, likely contributing to both delayed and potentially inappropriate diagnoses. There may be an increased prevalence of gastroparesis in hEDS and POTS populations, suggesting that future studies could incorporate gastric emptying assessments to differentiate gastroparesis from other functional gastrointestinal disorders.

However, it is crucial to acknowledge the limitations inherent in current study designs, such as the heterogeneity of data sources, limited longitudinal data, potential publication bias, and the underrepresentation of diverse populations. These limitations may impact the findings, and addressing them in future research would provide a more balanced and comprehensive understanding of these conditions and their interrelationships.
